# Surgical Aortic Mitral Curtain Replacement: Systematic Review and Metanalysis of Early and Long-Term Results

**DOI:** 10.3390/jcm10143163

**Published:** 2021-07-17

**Authors:** Ilaria Giambuzzi, Giorgia Bonalumi, Michele Di Mauro, Maurizio Roberto, Silvia Corona, Francesco Alamanni, Marco Zanobini

**Affiliations:** 1 IRCCS Centro Cardiologico Monzino, Department of Cardiovascular Surgery, 20100 Milan, Italy; giorgia.bonalumi@cardiologicomonzino.it (G.B.); maurizio.roberto@cardiologicomonzino.it (M.R.); silviacoro89@gmail.com (S.C.); francesco.alamanni@ccfm.it (F.A.); marco.zanobini@cardiologicomonzino.it (M.Z.); 2 DISCCO Department, University of Milan, 20100 Milan, Italy; 3 Heart and Vascular Centre, Cardio-Thoracic Surgery Unit, Maastricht University Medical Centre (MUMC), 9 Cardiovascular Research Institute Maastricht (CARIM), 6221 Maastricht, The Netherlands; mdimauro1973@gmail.com

**Keywords:** endocarditis, aortic mitral curtain, Commando procedure, UFO procedure

## Abstract

The Commando procedure is challenging, and aims to replace the mitral valve, the aortic valve and the aortic mitral curtain, when the latter is severely affected by pathological processes (such as infective endocarditis or massive calcification). Given the high complexity, it is seldomly performed. We aim to review the literature on early (hospitalization and up to 30 days) and long-term (at least 3 years of follow-up) results. Bibliographical research was performed on PubMed and Cochrane with a dedicated string. Papers regarding double valve replacement or repair in the context of aortic mitral curtain disease were included. The metaprop function was used to assess early survival and complications (pacemaker implantation, stroke and bleeding). Nine papers (540 patients, median follow-up 41 (IQR 24.5–51.5) months) were included in the study. Pooled proportion of early mortality, stroke, pacemaker implant and REDO for bleeding were, respectively 16.2%, 7.8%, 25.1% and 13.1%. The long-term survival rate ranged from 50% to 92.2%. Freedom from re-intervention was as high as 90.9% when the endocarditis was not the first etiology and 78.6% in case of valvular infection (one author had 100%). Freedom from IE recurrences reached 85% at 10 years. Despite the high mortality, the rates of re-intervention and infective endocarditis recurrences following the Commando procedure are satisfactory and confirm the need for an aggressive strategy to improve long-term outcomes.

## 1. Introduction

The aortic mitral curtain (AMC) is a complex structure contributing to the fibrous skeleton of the heart. It is located between the lateral and medial trigones of the mitral valve, connecting it to the left and non-coronary cusps of the aortic valve. Therefore, the AMC is related to the anterior leaflet of the mitral valve, the aortic valve cusps and the roof of the left atrium, which separates the mitral valve from the aortic root. When the AMC is involved in pathological processes, the “Commando” [1,2] (also called “UFO” procedure [3]) is performed (Supplementary File, Surgery). It was firstly described in 1976 [4]. This technique consists of aortic and mitral valve replacement and Left Ventricular Outflow Tract (LVOT) reconstruction with autologous or allograft pericardium. A variation, named Hemi-Commando [5,6], was proposed. It consists of replacing the aortic valve with a homograft, reconstructing the AMC and the anterior leaflet of the mitral valve. The mitral valve repair in such a context can be performed only in a less extensive disease, where at least part of the annulus and leaflets can be saved. Given the invasiveness of the procedure and the rarity of the AMC involvement, the procedure is rarely performed [7,8], and only few centers have published their results. First of all, the Commando is indicated when there is infective endocarditis [7,8,9,10,11], which, by invading the aortic and mitral valve, scavenges through the AMC, destroying it, with the formation of abscess/pseudo-aneurysm/fistula. Second, it is indicated in case of massive valvular calcification so that an “en bloc” resection of the AMC, the aortic and mitral valve is needed when the valves are stenotic or regurgitant [7,12,13]. Finally, the Commando procedure can also be used in case of double valve replacement complicated by unfavorable anatomy (small annuli). It allows to reconstruct the AMC in order to place a bigger prosthesis; in the case of a lack of tissue, anchoring the new prosthesis is particularly challenging, which is typical of REDO surgery [14,15].

Moreover, the early and late mortality rate is very high because of the poor general conditions of these patients and because of the invasiveness of the surgery itself. The aim of this systematic review is to summarize the evidence of the current literature on early (regarding mortality and complications during hospitalization) and long-term results (survival, new IE and re-intervention after at least 3 years) of the Commando procedure, when applied both to IE and to other pathological processes. We present the following article in accordance with the PRISMA reporting checklist.

## 2. Materials and Methods

### 2.1. Research

A PubMed research, following PRISMA guidelines [16] (Supplemental Table S1 and Supplemental Figure S1), was performed by two independent authors (I.G. and G.B.), using the string “(((endocarditis) AND ((reconstruction of the Aortomitral Fibrous Body) OR (aortic-mitral curtain) OR (Hemi-Commando operation) OR (Commando operation) OR (intervalvular fibrous body reconstruction) OR (UFO procedure) OR (LVOT reconstruction))”. Another search on Cochrane server was performed with a combination of search terms “Commando Procedure”, “UFO procedure”, “Mitro aortic reconstruction” and “Mitro aortic curtain endocarditis”; no inherent papers were found. No time limit was used in the research because of the few papers published on this subject.

The search (24 November 2020) included a total of 46 papers (Supplemental Table S1 and Supplemental Figure S1). Only English original articles that included a population of patients undergoing mitroaortic valve replacement or repair (Commando or Hemi-Commando) with AMC involvement were chosen.

Eleven papers were identified: one was excluded as the article was in Spanish, and the other one [8] was not included as the same authors published an updated series of patients few years later [17].

The quality of included studies was assessed using the Newcastle–Ottawa Scale for observational studies by two investigators independently (IG, GB) (Supplemental Table S2).

Therefore, a total of 9 papers (all retrospective, not randomized) were selected (Supplemental Table S3).

### 2.2. Data Extraction

Microsoft Office Excel (Microsoft, Redmond, WA, USA) was used for data extraction that was performed independently by two researchers (IG, GB). The following study characteristics were collected: age, year of publication, indications for procedure, rate of non-elective surgery, type of procedure, type of valve prostheses implanted and cardiopulmonary bypass (CPB) time.

### 2.3. Statistical Analysis and End-Points

Calculation of an overall proportion from studies reporting a single proportion was performed using a meta-analytic approach by means of metaprop function of “meta” package in the R STUDIO [18,19,20,21]. A logit-transformation was performed as suggested by Warton and Hui; to calculate confidence intervals (CIs) for individual study results, Clopper–Pearson approach was used; the inverse variance method was used for data pooling. Subgroup analysis was performed using random effect. DerSimonian–Laird estimator was used to estimate the between-study variance. Total proportion with 95% Cl was reported. Heterogeneity was reported as: low (I^2^ = 0–25%), moderate (I^2^ = 26–75%) and high (I^2^ > 75%). Funnel plot and Egger’s test were used for estimation of publication bias. Proportions were pooled for death, stroke, postoperative pacemaker (PM) implantation and reoperation for bleeding. Furthermore, some variables reported in the nine studies were tested to evaluate differences in terms of pooled proportions: age class (<55 vs. 55–59 vs. >59 years); year of publication; indications for procedure: only endocarditis vs. endocarditis/other indications; rate of non-elective surgery (<50% vs. 50–75% vs. >75%); only Commando vs. Commando or Hemi-Commando; type of valve prostheses implanted (mainly biological vs. mainly mechanical); range of CPB time (<2.5 h vs. 2.5–4.5 h vs. >4.5 h). Mainly biological or mechanical prostheses implanted was defined as rate >60%.

## 3. Results

All of the selected studies are retrospective, ranging from 7 years [10] to 29 years [11]. Five studies take into account the Hemi-Commando procedure [10,11,22,23] and, together with Davierwala PM et al. [17], they considered only patients with IE as etiology, while the other groups [7,12,15,24] also considered the other pathological processes (Supplemental Table S3). The main outcomes described by the authors are early (hospitalization up to 30 days) and late mortality (at least 3 years), the rate of reoperation and the rate of recurrence of infective endocarditis (Table 1). Surgical techniques are described in Supplemental Table S4. The total number of patients was 540, and the median follow-up was 41 (IQR 24.5–51.5) months.

### 3.1. Meta-Analysis

#### 3.1.1. Early Mortality

**Figure 1:**
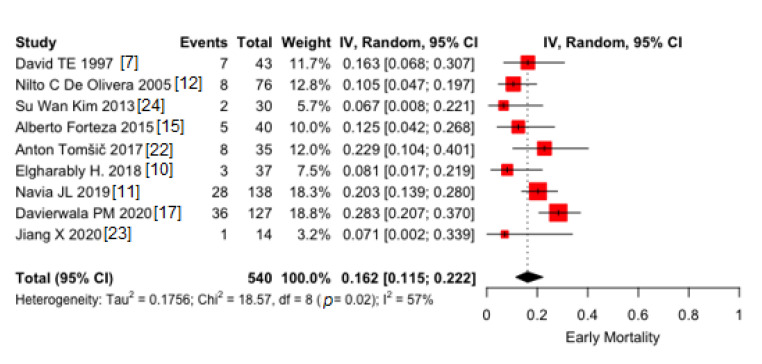
Pooled proportions of early deaths in 9 included studies. Black diamond was the pooled proportion. IV = inverse variance. Proportion is reported on X-axis.

Among the included nine studies, 540 patients, pooled proportion of early deaths was 16.2% (95% CI 11.5–22.2), (Figure 1). Moderate heterogeneity was found (I^2^ = 57%). Conversely, no publication bias was found (*p* = 0.10).

Early mortality was mainly due to technical surgical problems and multiorgan failure. When multivariate logistic regression was performed, S. aureus-positive blood culture, ejection fraction (EF) < 35% [17], older age (>60 years) [15], female sex and emergent surgery [11] were found as predictive factors for early mortality. The highest rate of death was presented by Davierwala et al. [17], and it was 28.3%. The lowest was described by Xuan Jiang et al. [23]. Of note, Davierwala et al. [17] presented older patients (whose mean age was 65.3 ± 12.9) when compared to Xuan Jiang et al. [23] (45.5 ± 13.0). Moreover, the Euroscore II by Davierwala was high (53.0 ± 25.2), but Xuan Jiang et al. [20] did not present data.

#### 3.1.2. Stroke

**Figure 2:**
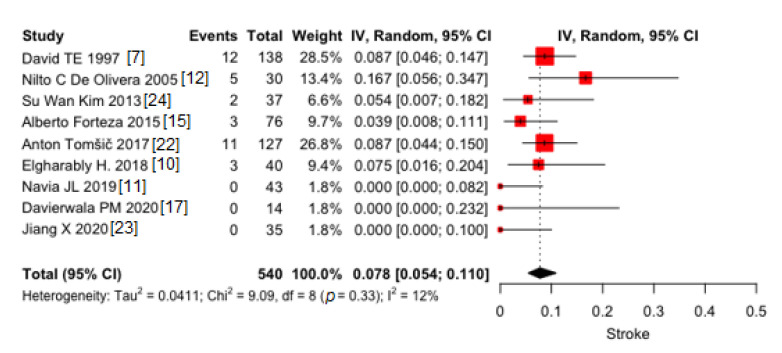
Pooled proportions of early strokes in 9 included studies. Black diamond was the pooled proportion. IV = inverse variance. Proportion is reported on X-axis.

Pooled proportion of early strokes was 7.8% (95% CI 5.4–11.0) (Figure 2). Very low heterogeneity was found (I^2^ = 12%). No publication bias was found (*p* = 0.08).

Differently from early mortality, most papers had similar rates of postoperative stroke. Su Wan Kim et al. [24] had the highest rate of stroke. It is in line with surgical times, which were indeed very long (CPB time was 297 ± 71 min), and their retrospective study was conducted previously than the studies of other authors with similar CPB times [17,22].

#### 3.1.3. PM Implant

**Figure 3:**
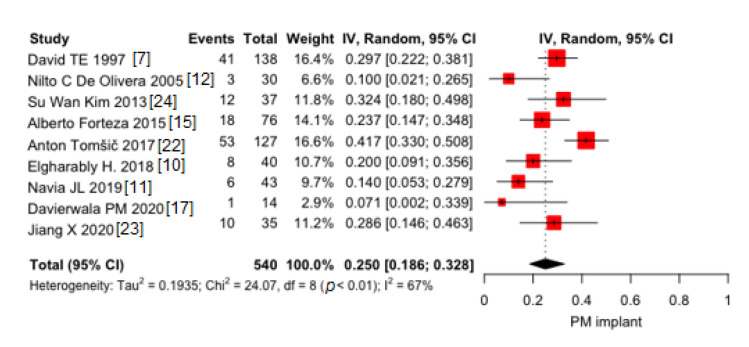
Pooled proportions of early pacemaker (PM) implant in 9 included studies. Black diamond was the pooled proportion. IV = inverse variance. Proportion is reported on X-axis.

Pooled proportion of early PM implants was 25.1% (95% CI 18.6–32.8) (Figure 3). Moderate heterogeneity was found (I^2^ = 67%). No publication bias was found (*p* = 0.07).

Moderate heterogeneity was expected in the rate of PM implant, first of all, because the papers span a long range of time and, second of all, because of the different indications to PM implantation that are adopted in hospitals.

#### 3.1.4. Redo for Bleeding

**Figure 4:**
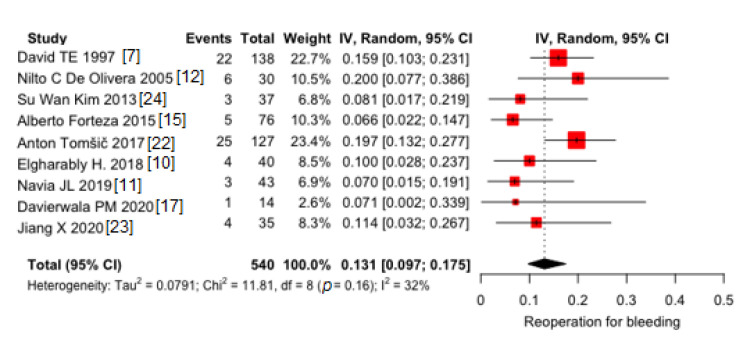
Pooled proportions of early reoperation for bleeding in 9 included studies. Black diamond was the pooled proportion. IV = inverse variance. Proportion is reported on X-axis.

Pooled proportion of early reoperation for bleeding was 13.1% (95% CI 9.6–17.5) (Figure 4). Moderate heterogeneity was found (I^2^ = 32%). No publication bias was found (*p* = 0.10).

As for PM implantation, REDO for bleeding also depends on hospital guidelines, and the indications for critical bleeding might change in different cardiac departments. Nevertheless, Davierwala et al. [17] and Navia JL [11] had the highest number of patients, and it might also be related to the higher percentages of post-operative bleeding.

#### 3.1.5. Modifier Variables

None of the investigated variables significantly impact the pooled proportion of both early deaths and strokes. The pooled proportion of early PM implant in the studies where indication to the surgical procedure was only the presence of endocarditis [10,11,17,22,23] was significantly higher (*p* = 0.0099) than in the other studies where extensive calcification and lack of tissues were also followed as indications [7,12,15,24], and they were, respectively, 32.4% (95% CI 24.7–41.2) vs. 18.8% (95% CI 13.5–25.7) (Supplemental Figure S2).

Similarly, the pooled rate of early PM implant was significantly higher (*p* = 0.0011) in four studies [10,11,17,22] where mainly biological prostheses were used versus five studies [7,12,15,23,24] where mainly mechanical prostheses were used; they were, respectively, 34.1% (95% CI 27.4–41.4) vs. 18.2% (95% CI 13.0–24.9) (Supplemental Figure S3).

Finally, in the three studies [7,12,23] where aortic clamping time lasted less than 2.5 h, pooled rate of reoperation for bleeding was significantly lower (6.8%, 95% CI 3.6–12.5) versus aortic clamping time between 2.5 and 4.5 h, and aortic clamping time > 4.5 h (*p* = 0.0472) (Supplemental Figure S4).

## 4. Discussion

The “Commando” procedure allows to reconstruct the AMC, and it is proposed for surgical treatment of extensive paravalvular disease, especially in the case of IE as etiology [7].

It is a complex procedure, which requires long CPB and aortic cross-clamping times. It requires detailed anatomical knowledge and a practical learning curve. Patients usually arrive with a deteriorated clinical condition, heart failure or septic shock, and they might have coagulopathies because of the infectious process. In the case of non-infective disease, they are usually older and with multiple co-pathologies (chronic kidney failure, lung disease, high NYHA class) [25]. Furthermore, many of them have already undergone previous cardiac surgery, enhancing the surgical risk. Therefore, in the case of an AMC destructive process, on the one hand, it is important to guarantee a satisfying long-term survival and, on the other hand, to lower early mortality and post-operative complications.

Indeed, our results showed high mortality (pooled proportion of 16.2%) and post-operative complications (pooled proportions of stroke, PM implantation and REDO for bleeding were, respectively, 7.8%, 25% and 13.1%). The long-term survival, freedom from re-intervention and freedom from re-infection reached, respectively, 57 ± 5%, 84 ± 5% and 82 ± 4%.

Therefore, both early mortality (pooled proportion is 16.2%) and late mortality (long-term survival might be as low as 37% [11]) are high, probably due to a combination of multiple factors, such as the poor pre-operative clinical condition, the challenging surgery, the long post-operative course and IE recurrence. Nevertheless, Darvierwala PM et al. [8] showed that, after gaining experience, the highest mortality is in the first 90 days and, after excluding those deaths, the rates of survival are higher than 70% [17]. Therefore, it is clear that the most delicate period is the immediate post-operative course [22]. It is then advisable to perform this kind of surgery in specialized centers that have a high level of expertise in the endocarditis heart team. Anyway, the hospital mortality for patients operated for IE ranges from 15% to 30% [9,26,27,28], which is in line with the aforementioned results for the Commando procedure.

Post-operative complications included stroke, PM implantation and reoperation for bleeding. The pooled rate of stroke is 7.8%, very high when compared to stroke after cardiac surgery [29]. It might be explainable by micro-embolization of surgical debris and calcium after surgery; moreover, aortic clamping time and CPB time, known risk factors for stroke [30], are long in all studies (which also explains the high rate of reoperation for bleeding [31]). The high rate [32] of PM implantation (25%) is easily explainable by the proximity of the atrioventricular node to the structures involved in the Commando/Hemi-Commando surgery. In our study, biological prostheses had a higher rate of PM implantation, which is not described by current literature [33,34]. It might be due to the fact that, in the Commando procedure, where the annular tissue is severely diseased, the pivot of the strut of the biological prosthesis could enhance the pressure on the atrioventricular node, making them a variable associated with an increased risk of PM implantation. Further studies are needed to address this result.

Long-term mortality is not much different when compared to IE patients treated with the standard surgery, which ranges from 60% to 70% at 5 years [35,36,37]. Croon SI et al. showed a survival rate of 55% at 10 years [38]. However, it has been shown that the hospital mortality and survival were similar in a matched cohort of patients [2]. Indeed, overall survival is stable after the first year of surgery. Jiang X et al. [23] had outstanding results regarding survival and rate of re-intervention since no patient required redo surgery. Furthermore, the patients who underwent Commando for reasons other than IE had good survival rates even after 10 years [7,12,15,24].

An invasive surgery such as the Commando procedure guarantees, in case of IE, complete debridement of the infective material and, in case of the other described pathological process, a lower risk of prosthesis dehiscence, paravalvular leak and patient–prosthesis mismatch, which are all possible cause of re-intervention [39,40]. De Olivera NC et al. [12] described every case of reoperation after the Commando procedure. In their series, 12 patients (15.79%) required a re-intervention (among them, three patients had multiple re-interventions). Their decision was based upon prosthetic valve endocarditis in seven patients, four valve dehiscences/degenerations, four patch tears/dehiscences and one paravalvular leak. Their freedom from re-intervention was 83 ± 5% and 73 ± 7% at 5 and 10 years, respectively. Among the re-operated patients, three (25%) died. In any case, they reduced surgical failure together as they increased surgical experience.

Regarding re-infection, the type of prosthesis plays a key role. Indeed, homografts have a lower rate of re-infection when compared to biological and mechanical prostheses [41,42], probably because of the strong inflammatory reaction elicited by the stent of the prosthesis. Nevertheless, in case of extensive IE involving AMC, there are only a few cases treated with implantation of double homograft (aortic and mitral) [43,44] and, moreover, even if the rate of re-infection after homograft is lower [45], the re-interventions in case of structural deterioration are very challenging and satisfying results are obtained only in dedicated high volume centers [46].

It is difficult to compare the Commando operation to a less aggressive strategy in terms of rate of re-intervention, not only because of the rarity of the disease, but also because patients requiring it are morbid and present an extensive involvement and destruction of cardiac structures. It is also widely recognized that an aggressive approach for complete debridement should be employed whenever possible by experienced surgeons [12]. The need for extensive debridement is also confirmed by the reported high freedom from IE recurrences, reaching 94.7% at 5 years [24]. Such a high rate of freedom from IE is extremely satisfactory, as the initial disease is very extensive and, therefore, more prone to develop re-infection [47]. On the contrary, Navia JL et al. [11] showed initial higher freedom from recurrences in the Hemi-Commando group, but, at 8 years, the Hemi-Commando group kept having IE recurrences, while the Commando group was stable at around 70% of freedom from IE. The difference might be due to the lower number of patients in the Hemi-Commando group available for follow-up after 8 years, so further studies are needed to clarify this point. David TE et al. [48] studied a group of patients with paravalvular abscess (without AMC involvement) who needed annulus reconstruction before implantation of the new prosthesis. This population might be suited to be compared to patients who underwent Commando procedures, since the surgery was often urgent/emergent (65%) and the involvement of cardiac structure was more extensive than classical valve endocarditis. The 10-year survival was 57 ± 5%, the freedom from recurrent infective endocarditis was 82 ± 4% at 10 years and freedom from reoperation at 10 years was 84 ± 5%. Their results are comparable to the ones reported on the series of Commando patients, showing that the Commando surgery guarantees a result comparable to patients with less extensive disease.

### Limitations

The most important limitations of these studies regard the retrospective nature and the paucity of included patients, giving, therefore, a risk of bias. The heterogeneity derives from the clinical differences of these patients, which are all very complex and hard to properly prepare pre-operatively because of the urgent nature of the disease.

Moreover, most of them are tertiary centers; therefore patients were nationally and internationally referred, which explains why there are some lost to follow-up.

## 5. Conclusions

Pathologies involving the AMC carry a high surgical risk, and, in some cases, an invasive surgery such as the Commando procedure might be the only solution. The high mortality rate is due to both the etiology and the impact of the procedure. Nevertheless, in such a severe pathology, the only way to lower the risk of recurrence and to give a chance to recover, is to have an experienced surgeon who, after performing a deep debridement by disassembling the heart, can also reconstruct it.

## Figures and Tables

**Table 1:** Authors, aims, results.

First Author	Tot Patients and FUP	Survival	Re-Operation	Recurrent IE
David T.E. [7]	43 patients Mean follow-up 38 ± 29 months	7 intrahospital deaths (16%) 6 late deaths Actuarial survival 6 years 56 ± 6%	2 (early IE)	2 patients early IE 1 patient late IE
Nilto C. De Oliveira [12]	76 patients Mean of 47± 47 months	8 operative deaths (10%) 18 late deaths (24%) Overall survival 5 years 71 ± 6% 10 years 50% ± 9%	12 patients Freedom from any re-operation 5 years 83 ± 5% 10 years 73 ± 7%	7 patients Freedom from IE 5 years 85% ± 5% 10 years 85% ± 5%
Su Wan Kim [24]	30 patients Mean follow-up 50.6 ± 43.5 months	2 hospital deaths (6.7%) 5 late deaths Survival rate in IE patients 1 year 80.8% 5 year 74.6% (5 late deaths) Survival rate in non-IE patients 1 year 87.5% 5 years 87.5% *p*-value 0.76	3 patients (2 IE, 1 heart transplant) Freedom from REDO in IE patients 1 year 95.5% 5 year 84.8%	1 patient Freedom from IE in IE patients at 1 year 94.7% 5 years 94.7%
Alberto Forteza [15]	40 patients Mean follow-up was 53 ± 8 months	Hospital mortality rate 22.2% (5 patients) Survival rate group A 1 year 65.4% 5 year 57.7% 10 year 50% Group B 1 year 92.9% 5 years 85.7% 10 years 78.6%	5 patients Freedom from re-operation group A 1 year 92.3% 5 year 84.6% 10 year 76.0% Group B 1 year 90.9% 5 years 90.9% 10 years 90.9%	4 patients
Anton Tomšič [22]	35 patients Median follow-up 29.8 months	8 early deaths (23%) 5 follow-up deaths Overall survival 1 year: 69.4% 2 years: 65.3% 5 years: 65.3%	7 patients Freedom from REDO 1 year: 84.6% 2 years: 78.9% 5 years: 51.2%	2 patients
Elgharably H. [10]	37 patients Mean follow-up 581 ± 729 days	3 hospital deaths (8%) 2 post-discharge deaths (5%) Overall survival 1 year: 91% 3 years: 82%	1 for aortic pseudoaneurysm (3%)	1 patient (3%)
Navia J.L. [11]	138 patients Mean follow-up 41 ± 5.9 months	28 hospital deaths (20%); 21 Commando (24%), 7 Hemi-Commando (13%) Overall survival 1 year 67% 5 year 48% 10 year 37% Commando survival 1 year 60% 5 year 40% 10 year 35% Hemi-Commando survival 1 year 80% 5 year 64% 10 year 57% No significant difference	Commando: 19 (38%) Hemi-Commando: 4 (12%) Freedom from REDO Commando 1 year 83% 5 year 63% 8 year 50% Freedom from REDO Hemi-Commando 1 year 87% 5 year 74% 8 year 55%	Commando: 13 (26%); Hemi-Commando: 4 (12%) Freedom from recurrent IE Commando 1 year 87% 5 years 77% 8 years 70% Freedom from recurrent IE Hemi-Commando 1 year 95% 5 years 82% 8 years 41% No significant difference
Davierwala PM [17]	127 patients Mean follow-up 477.5 ± 799.2 months	Early deaths (30 days): 36 (28.3%); 90 days 47 (37%) Late deaths (follow-up > 90 days): 13 (10.2%) Survival rates 3 years 45.3 ± 5.1 5 years 41.8 ± 5.8%,	9 patients (7 IE, 2 miscellaneous) Freedom from REDO 5 years 85.1 ± 5.7%	7 patients
Jiang X [23]	14 patients Mean follow-up 18.9 ± 12.2 months	Early deaths (60 days): 1 No deaths at follow-up	No re-intervention	No IE

FUP: follow-up; IE: Infective endocarditis.

## Supplementary Materials

The following are available online at https://www.mdpi.com/article/10.3390/jcm10143163/s1, Supplemental Table S1—Prisma checklist; Supplemental Table S2—Newcastle–Ottawa Scale; Surgery—Description of the technique; Supplemental Table S3—Authors, year, papers and total number of patients; Supplemental Table S4—Authors and description of the techniques; Supplemental Figure S1—Prisma flowchart; Supplemental Figure S2—Early pacemaker implant by indication to surgery; Supplemental Figure S3—Early pacemaker implant by type of prosthesis; Supplemental Figure S4—Early reoperation for bleeding by cardiopulmonary bypass time.
