# Supplementary material for: Surgical Aortic Mitral Curtain Replacement: Systematic Review and Metanalysis of Early and Long-Term Results
Source: J Clin Med. 2021 Jul 17;10(14):3163. doi: 10.3390/jcm10143163 (PMC8305575; doi:10.3390/jcm10143163)
Supplement: Supplementary file 1 [file jcm-10-03163-s001.zip › jcm-1269422-supplementary.pdf]

Supplemental Table S1. Prisma Checklist.

Section/topic	#	Checklist item	Reported on page #
**TITLE**			
Title	1	Identify the report as a systematic review, meta-analysis, or both.	1
**ABSTRACT**			
Structured summary	2	Provide a structured summary including, as applicable: background; objectives; data sources; study eligibility criteria, participants, and interventions; study appraisal and synthesis methods; results; limitations; conclusions and implications of key findings; systematic review registration number.	1
**INTRODUCTION**			
Rationale	3	Describe the rationale for the review in the context of what is already known.	1 – 2
Objectives	4	Provide an explicit statement of questions being addressed with reference to participants, interventions, comparisons, outcomes, and study design (PICOS).	2
**METHODS**			
Protocol and registration	5	Indicate if a review protocol exists, if and where it can be accessed (e.g., Web address), and, if available, provide registration information including registration number.	-
Eligibility criteria	6	Specify study characteristics (e.g., PICOS, length of follow-up) and report characteristics (e.g., years considered, language, publication status) used as criteria for eligibility, giving rationale.	3
Information sources	7	Describe all information sources (e.g., databases with dates of coverage, contact with study authors to identify additional studies) in the search and date last searched.	3
Search	8	Present full electronic search strategy for at least one database, including any limits used, such that it could be repeated.	3
Study selection	9	State the process for selecting studies (i.e., screening, eligibility, included in systematic review, and, if applicable, included in the meta-analysis).	3
Data collection process	10	Describe method of data extraction from reports (e.g., piloted forms, independently, in duplicate) and any processes for obtaining and confirming data from investigators.	3
Data items	11	List and define all variables for which data were sought (e.g., PICOS, funding sources) and any assumptions and simplifications made.	3
Risk of bias in individual studies	12	Describe methods used for assessing risk of bias of individual studies (including specification of whether this was done at the study or outcome level), and how this information is to be used in any data synthesis.	3 – 6 supp.materi al
Summary measures	13	State the principal summary measures (e.g., risk ratio, difference in means).	3

Synthesis of results	14	Describe the methods of handling data and combining results of studies, if done, including measures of consistency (e.g., I^2) for each meta-analysis.	3
Risk of bias across studies	15	Specify any assessment of risk of bias that may affect the cumulative evidence (e.g., publication bias, selective reporting within studies).	3 -6
Additional analyses	16	Describe methods of additional analyses (e.g., sensitivity or subgroup analyses, meta-regression), if done, indicating which were pre-specified.	3
**RESULTS**			
Study selection	17	Give numbers of studies screened, assessed for eligibility, and included in the review, with reasons for exclusions at each stage, ideally with a flow diagram.	3–Supp. table
Study characteristics	18	For each study, present characteristics for which data were extracted (e.g., study size, PICOS, follow-up period) and provide the citations.	3-4
Risk of bias within studies	19	Present data on risk of bias of each study and, if available, any outcome level assessment (see item 12).	3–supp. material
Results of individual studies	20	For all outcomes considered (benefits or harms), present, for each study: (a) simple summary data for each intervention group (b) effect estimates and confidence intervals, ideally with a forest plot.	7-8-9-10
Synthesis of results	21	Present results of each meta-analysis done, including confidence intervals and measures of consistency.	6
Risk of bias across studies	22	Present results of any assessment of risk of bias across studies (see Item 15).	3–supp. Material
Additional analysis	23	Give results of additional analyses, if done (e.g., sensitivity or subgroup analyses, meta-regression [see Item 16]).	-
**DISCUSSION**			
Summary of evidence	24	Summarize the main findings including the strength of evidence for each main outcome; consider their relevance to key groups (e.g., healthcare providers, users, and policy makers).	11-12
Limitations	25	Discuss limitations at study and outcome level (e.g., risk of bias), and at review-level (e.g., incomplete retrieval of identified research, reporting bias).	12
Conclusions	26	Provide a general interpretation of the results in the context of other evidence, and implications for future research.	12
**FUNDING**			
Funding	27	Describe sources of funding for the systematic review and other support (e.g., supply of data); role of funders for the systematic review.	12

PRISMA 2009 Checklist and flow diagram

From: Moher D, Liberati A, Tetzlaff J, Altman DG, The PRISMA Group (2009). Preferred Reporting Items for Systematic Reviews and Meta-Analyses: The PRISMA Statement. PLoS Med 6(7): e1000097. doi:10.1371/journal.pmed1000097

For more information, visit: www.prisma-statement.org.

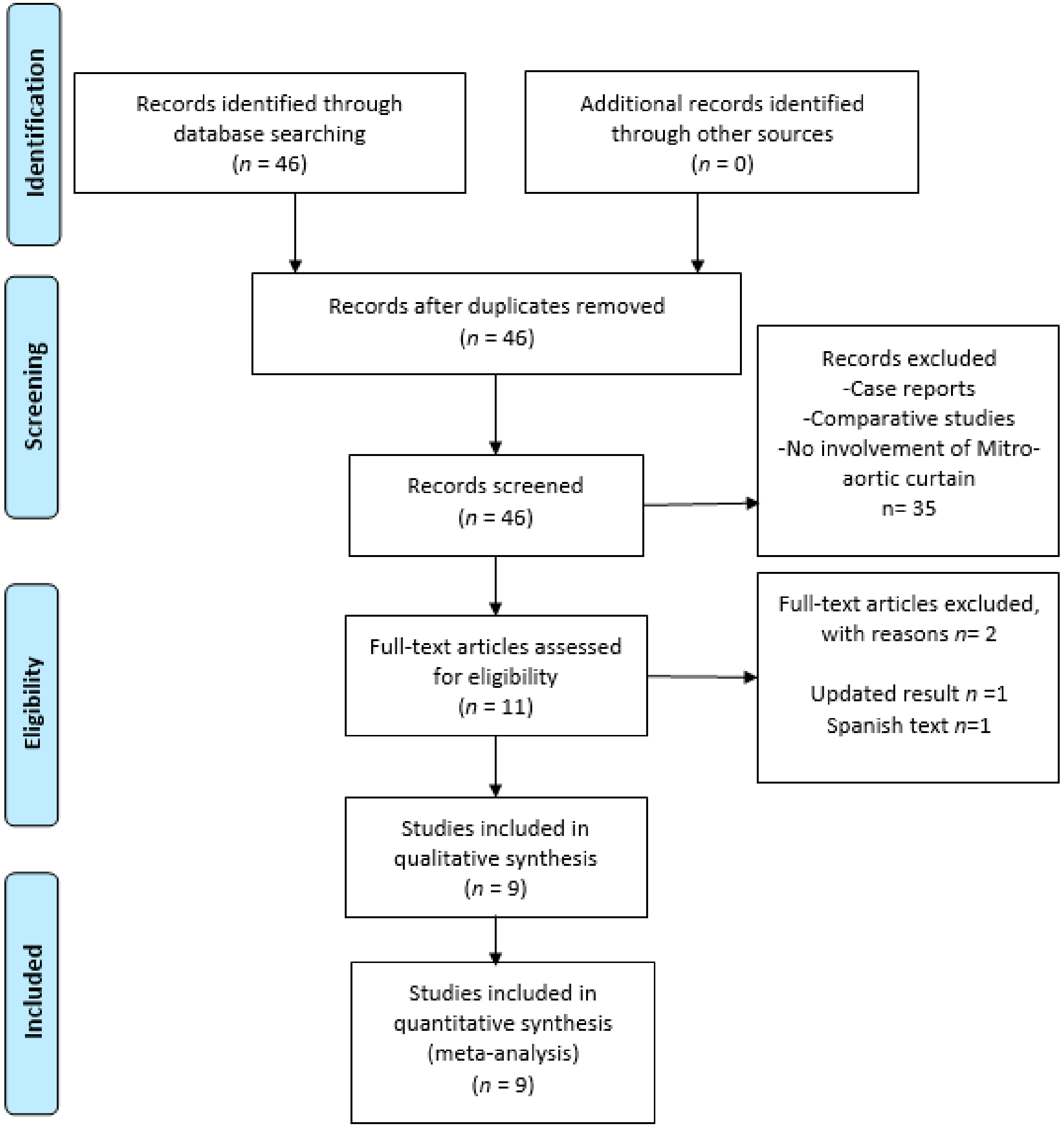

Supplemental Figure S1. Prisma flowchart.

Supplemental Table S2. Newcastle-Ottawa Scale.

**Study: David TE 1997 [7]**							
**Selection**				**Comparability**	**Outcome**		
**Representativeness of the exposed cohort**	**Selection of the non-exposed cohort**	**Ascertainment of exposure**	**Demonstration that outcome of interest was not present at start of study**	**Comparability of cohorts on the basis of the design or analysis controlled for confounders**	**Assessment of outcome**	**Was follow-up long enough for outcomes to occur**	**Adequacy of follow-up of cohorts**
a) Truly representative ×<br>b) Somewhat representative ×<br>c) Selected group<br>d) No description of the derivation of the cohort	a) Drawn from the same community as the exposed cohort ×<br>b) Drawn from a different source<br>c) No description of the derivation of the non exposed cohort	a) Secure record (e.g., surgical record) ×<br>b) Structured interview ×<br>c) Written self report<br>d) No description<br>e) Other	a) Yes ×<br>b) No	a) The study controls for age, sex and marital status ×<br>b) Study controls for other factors (urgency, euroscore, age) ×<br>c) Cohorts are not comparable on the basis of the design or analysis controlled for confounders	a) Independent blind assessment ×<br>b) Record linkage ×<br>c) Self report<br>d) No description<br>e) Other	a) Yes ×<br>b) No	a) Complete follow up- all subject accounted for ×<br>b) Subjects lost to follow up unlikely to introduce bias- number lost less than or equal to 20% or description of those lost suggested no different from those followed. ×<br>c) Follow up rate less than 80% and no

							description of those lost<br>d) No statement
**Study: Nilto C. De Oliveira et al. 2005 [12]**							
Selection ××× ; Comparability / ; Outcome							
**Selection**				**Comparability**	**Outcome**		
**Representativeness of the exposed cohort**	**Selection of the non-exposed cohort**	**Ascertainment of exposure**	**Demonstration that outcome of interest was not present at start of study**	**Comparability of cohorts on the basis of the design or analysis controlled for confounders**	**Assessment of outcome**	**Was follow-up long enough for outcomes to occur**	**Adequacy of follow-up of cohorts**
a) Truly representative ×<br>b) Somewhat representative ×<br>c) Selected group<br>d) No description of the derivation of the cohort	a) Drawn from the same community as the exposed cohort ×<br>b) Drawn from a different source<br>c) No description of the derivation of the non exposed cohort	a) Secure record (e.g., surgical record) ×<br>b) Structured interview ×<br>c) Written self report<br>d) No description<br>e) Other	a) Yes ×<br>b) No	a) The study controls for age, sex and marital status ×<br>b) Study controls for other factors (urgency, euroscore, age) ×<br>c) Cohorts are not comparable on the basis of the design or analysis controlled for confounders	a) Independent blind assessment ×<br>b) Record linkage ×<br>c) Self report<br>d) No description<br>e) Other	a) Yes ×<br>b) No	a) Complete follow up- all subject accounted for ×<br>b) Subjects lost to follow up unlikely to introduce bias- number lost less than or equal to 20% or description of those lost suggested no different from those followed. ×<br>c) Follow up rate less than 80% and no

							description of those lost<br>d) No statement
××. Selection ×××; Comparability /; Outcome ×××							
**Study: Su Wan Kim et al. 2013 [24]**							
**Selection**				**Comparability**	**Outcome**		
**Representativeness of the exposed cohort**	**Selection of the non-exposed cohort**	**Ascertainment of exposure**	**Demonstration that outcome of interest was not present at start of study**	**Comparability of cohorts on the basis of the design or analysis controlled for confounders**	**Assessment of outcome**	**Was follow-up long enough for outcomes to occur**	**Adequacy of follow-up of cohorts**
a) Truly representative ×<br>b) Somewhat representative ×<br>c) Selected group<br>d) No description of the derivation of the cohort	a) Drawn from the same community as the exposed cohort ×<br>b) Drawn from a different source<br>c) No description of the derivation of the non exposed cohort	a) Secure record (e.g., surgical record) ×<br>b) Structured interview ×<br>c) Written self report<br>d) No description<br>e) Other	a) Yes ×<br>b) No	a) The study controls for age, sex and marital status ×<br>b) Study controls for other factors (urgency, euroscore, age) ×<br>c) Cohorts are not comparable on the basis of the design or analysis controlled for confounders	a) Independent blind assessment ×<br>b) Record linkage ×<br>c) Self report<br>d) No description<br>e) Other	a) Yes ×<br>b) No	a) Complete follow up- all subject accounted for ×<br>b) Subjects lost to follow up unlikely to introduce bias- number lost less than or equal to 20% or description of those lost suggested no different from those followed. ×<br>c) Follow up rate less than 80% and no description of those lost<br>d) No statement
**Study: Alberto Forteza et al. 2015 [15]**							
**Selection**				**Comparability**	**Outcome**		

**Representativeness of the exposed cohort**	**Selection of the non-exposed cohort**	**Ascertainment of exposure**	**Demonstration that outcome of interest was not present at start of study**	**Comparability of cohorts on the basis of the design or analysis controlled for confounders**	**Assessment of outcome**	**Was follow-up long enough for outcomes to occur**	**Adequacy of follow-up of cohorts**
a) Truly representative × b) Somewhat representative × c) Selected group d) No description of the derivation of the cohort	a) Drawn from the same community as the exposed cohort × b) Drawn from a different source c) No description of the derivation of the non exposed cohort	a) Secure record (e.g., surgical record) × b) Structured interview × c) Written self report d) No description e) Other	a) Yes × b) No	a) The study controls for age, sex and marital status × b) Study controls for other factors (urgency, euroscore, age) × c) Cohorts are not comparable on the basis of the design or analysis controlled for confounders	a) Independent blind assessment × b) Record linkage × c) Self report d) No description e) Other	a) Yes × b) No	a) Complete follow up- all subject accounted for × b) Subjects lost to follow up unlikely to introduce bias- number lost less than or equal to 20% or description of those lost suggested no different from those followed. × c) Follow up rate less than 80% and no description of those lost d) No statement
Selection ×××; Comparability /; Outcome ××							
**Study: Anton Tomšič et al. 2017 [22]**							
**Selection**				**Comparability**	**Outcome**		

Representativeness of the exposed cohort	Selection of the non-exposed cohort	Ascertainment of exposure	Demonstration that outcome of interest was not present at start of study	Comparability of cohorts on the basis of the design or analysis controlled for confounders	Assessment of outcome	Was follow-up long enough for outcomes to occur	Adequacy of follow-up of cohorts
a) Truly representative ×<br>b) Somewhat representative ×<br>c) Selected group<br>d) No description of the derivation of the cohort	a) Drawn from the same community as the exposed cohort ×<br>b) Drawn from a different source<br>c) No description of the derivation of the non exposed cohort	a) Secure record (e.g., surgical record) ×<br>b) Structured interview ×<br>c) Written self report<br>d) No description<br>e) Other	a) Yes ×<br>b) No	a) The study controls for age, sex and marital status ×<br>b) Study controls for other factors (urgency, euroscore, age) ×<br>c) Cohorts are not comparable on the basis of the design or analysis controlled for confounders	a) Independent blind assessment ×<br>b) Record linkage ×<br>c) Self report<br>d) No description<br>e) Other	a) Yes ×<br>b) No	a) Complete follow up- all subject accounted for ×<br>b) Subjects lost to follow up unlikely to introduce bias- number lost less than or equal to 20% or description of those lost suggested no different from those followed. ×<br>c) Follow up rate less than 80% and no description of those lost<br>d) No statement
Selection ×××; Comparability /; Outcome ××							
**Study: Elgharably H. et al. 2018 [10]**							
**Selection**				**Comparability**	**Outcome**		

**Representativeness of the exposed cohort**	**Selection of the non-exposed cohort**	**Ascertainment of exposure**	**Demonstration that outcome of interest was not present at start of study**	**Comparability of cohorts on the basis of the design or analysis controlled for confounders**	**Assessment of outcome**	**Was follow-up long enough for outcomes to occur**	**Adequacy of follow-up of cohorts**
a) Truly representative ×<br>b) Somewhat representative ×<br>c) Selected group<br>d) No description of the derivation of the cohort	a) Drawn from the same community as the exposed cohort ×<br>b) Drawn from a different source<br>c) No description of the derivation of the non exposed cohort	a) Secure record (e.g., surgical record) ×<br>b) Structured interview ×<br>c) Written self report<br>d) No description<br>e) Other	a) Yes ×<br>b) No	a) The study controls for age, sex and marital status ×<br>b) Study controls for other factors (urgency, euroscore, age) ×<br>c) Cohorts are not comparable on the basis of the design or analysis controlled for confounders	a) Independent blind assessment ×<br>b) Record linkage ×<br>c) Self report<br>d) No description<br>e) Other	a) Yes ×<br>b) No	a) Complete follow up- all subject accounted for ×<br>b) Subjects lost to follow up unlikely to introduce bias- number lost less than or equal to 20% or description of those lost suggested no different from those followed. ×<br>c) Follow up rate less than 80% and no description of those lost<br>d) No statement
Selection ×××; Comparability /; Outcome ×							
**Study: Navia JL et al. 2019 [11]**							
**Selection**				**Comparability**	**Outcome**		

**Representativeness of the exposed cohort**	**Selection of the non-exposed cohort**	**Ascertainment of exposure**	**Demonstration that outcome of interest was not present at start of study**	**Comparability of cohorts on the basis of the design or analysis controlled for confounders**	**Assessment of outcome**	**Was follow-up long enough for outcomes to occur**	**Adequacy of follow-up of cohorts**
a) Truly representative × b) Somewhat representative × c) Selected group d) No description of the derivation of the cohort	a) Drawn from the same community as the exposed cohort × b) Drawn from a different source c) No description of the derivation of the non exposed cohort	a) Secure record (e.g., surgical record) × b) Structured interview × c) Written self report d) No description e) Other	a) Yes × b) No	a) The study controls for age, sex and marital status × b) Study controls for other factors (urgency, euroscore, age) × c) Cohorts are not comparable on the basis of the design or analysis controlled for confounders	a) Independent blind assessment × b) Record linkage × c) Self report d) No description e) Other	a) Yes × b) No	a) Complete follow up- all subject accounted for × b) Subjects lost to follow up unlikely to introduce bias- number lost less than or equal to 20% or description of those lost suggested no different from those followed. × c) Follow up rate less than 80% and no description of those lost d) No statement
Selection ×××; Comparability /; Outcome ××							
**Study: Davierwala PM 2020 [17]**							
**Selection**				**Comparability**	**Outcome**		

**Representativeness of the exposed cohort**	**Selection of the non-exposed cohort**	**Ascertainment of exposure**	**Demonstration that outcome of interest was not present at start of study**	**Comparability of cohorts on the basis of the design or analysis controlled for confounders**	**Assessment of outcome**	**Was follow-up long enough for outcomes to occur**	**Adequacy of follow-up of cohorts**
a) Truly representative ×<br>b) Somewhat representative ×<br>c) Selected group<br>d) No description of the derivation of the cohort	a) Drawn from the same community as the exposed cohort ×<br>b) Drawn from a different source<br>c) No description of the derivation of the non exposed cohort	a) Secure record (e.g., surgical record) ×<br>b) Structured interview ×<br>c) Written self report<br>d) No description<br>e) Other	a) Yes ×<br>b) No	a) The study controls for age, sex and marital status ×<br>b) Study controls for other factors (urgency, euroscore, age) ×<br>c) Cohorts are not comparable on the basis of the design or analysis controlled for confounders	a) Independent blind assessment ×<br>b) Record linkage ×<br>c) Self report<br>d) No description<br>e) Other	a) Yes ×<br>b) No	a) Complete follow up- all subject accounted for ×<br>b) Subjects lost to follow up unlikely to introduce bias- number lost less than or equal to 20% or description of those lost suggested no different from those followed. ×<br>c) Follow up rate less than 80% and no description of those lost<br>d) No statement
Selection ×××; Comparability /; Outcome ×××							
**Study: Jiang X et al. 2020 [23]**							
**Selection**				**Comparability**	**Outcome**		

Representativeness of the exposed cohort	Selection of the non-exposed cohort	Ascertainment of exposure	Demonstration that outcome of interest was not present at start of study	Comparability of cohorts on the basis of the design or analysis controlled for confounders	Assessment of outcome	Was follow-up long enough for outcomes to occur	Adequacy of follow-up of cohorts
a) Truly representative × b) Somewhat representative × c) Selected group d) No description of the derivation of the cohort	a) Drawn from the same community as the exposed cohort × b) Drawn from a different source c) No description of the derivation of the non exposed cohort	a) Secure record (e.g., surgical record) × b) Structured interview × c) Written self report d) No description e) Other	a) Yes × b) No	a) The study controls for age, sex and marital status × b) Study controls for other factors (urgency, euroscore, age) × c) Cohorts are not comparable on the basis of the design or analysis controlled for confounders	a) Independent blind assessment × b) Record linkage × c) Self report d) No description e) Other	a) Yes × b) No	a) Complete follow up- all subject accounted for × b) Subjects lost to follow up unlikely to introduce bias- number lost less than or equal to 20% or description of those lost suggested no different from those followed. × c) Follow up rate less than 80% and no description of those lost d) No statement
Selection ×××; Comparability /; Outcome ×××							

Author	Score	Selection	Comparability	Outcome
David TE [7]	5	×××	-	××
Nilto C. De Oliveira[12]	6	×××	-	×××

Su Wan Kim [24]	6	×××	-	×××
Alberto Forteza [15]	5	×××	-	××
Anton Tomšič [22]	5	×××	-	××
Elgharably H. [10]	4	×××	-	×
Navia JL [11]	5	×××	-	××
Davierwala PM [17]	6	×××	-	×××
Jiang X [23]	6	×××	-	×××

SURGERY [1]

The first step is the correct exposure of the cardiac chamber. An oblique aortotomy, towards the base of the non-coronary sinus, is performed. Then there can be a left atriotomy connecting the right superior pulmonary vein to the anterior mitral leaflet or a modification of the bi-atrial trans-septal opening by Guiraudon [26] in case of the tricuspid valve involvement.

The aortic valve is then removed and the anterior mitral leaflet is excised together with AMC. The left ventricle is exposed in a triangular shape, showing the posterior leaflet of the mitral valve and the aortic annulus. Then sutures for the mitral prosthesis are passed through the posterior annulus. At this point the AMC is reconstructed. It can be performed using the "double patch technique", where a single patch is sutured, U folded on the width of the lateral-to-medial trigon, where the mitral prosthesis will be sewn. The posterior part of the patch reconstructs the left atrial dome and the anterior part is used to reconstruct the non-coronary sinus of the aortic root, which can also be used to anchor the aortic valvular conduit. Reconstruction can also be performed using a "single patch technique". A triangle patch is used to

reconstruct the left atrial dome, and as a result the aortic valvular conduit is directly sutured to mitral valve prosthesis and patch.

The Hemi commando has been proposed in case of aortic root replacement and mitral valve disease involving only the anterior leaflet. A homo/allograft is then needed to replace "en bloc" the aortic root, the aortic valve and the anterior leaflet. A lower degree of damage of the mitral valve, involving only the anterior leaflet, is fundamental to consider the hemi-commando, being the mitral valve only repaired. A homo/allograft aortic conduit with anterior mitral leaflet is positioned as a unit, and finally a mitral valve ring annuloplasty is performed.

[1] https://mmcts.org/tutorial/45 (date last accessed 29/6/2021)

[2] Guiraudon, G.M.; Ofiesh, J.G.; Kaushik, R. Extended vertical transatrial septal approach to the mitral valve. Ann. Thorac. Surg. 1991, 52, 1058–60; discussion 1060–2, doi:10.1016/0003-4975(91)91281-y.

Supplemental Table S3 Authors, year, papers and total number of patients

First author	Journal	Year	Type of study	N° of patients	Indications
David TE[7]	J Thorac Cardiovasc Surg	1997	Retrospective, 1985 – 1996	14 (32,55%)	IE
			43 patients	9 (20,93%)	Extensive calcification
					Lack of tissue to anchor the prosthesis
				10 (23,26%)	Small annuli
				10 (23,26%)	
Nilto C. De Oliveira[12]	J thorac cardiovasc Surg	2005	Retrospective, 1985 - 2002	15 (19,74%)	IE
			76 patients	24 (31,57%)	Extensive calcification
					Lack of tissue to anchor the prosthesis
				17 (22,37%)	Prevent patient-prosthesis mismatch
				20 (26,32%)	
Su Wan	Ann thorac surg	2013	Retrospective, 1997-2010	22 (73,3%)	IE

Kim[24]			30 patients	5 (16,7%)<br><br><br>3 (10%)	Prevent patients-prosthesis mismatch<br>Severe Calcification
Alberto Forteza[15]	Ann Thorac Surg	2015	Retrospective, 1997-2014<br>40 patients	26 (65%)<br>14 (35%)	IE<br>Calcification/ Lack of tissue to anchor the prosthesis
Anton Tomšič[22]	Eur J Cardiothorac Surg	2017	Retrospective, 2004 – 2015<br>35 patients	35	IE
Elgharably H.[10]	Eur J Cardiothorac Surg	2018	Retrospective, 2010-2017<br>37 patients	37	IE
Navia JL[11]	Ann Thorac Surg	2019	Retrospective, 1988-2017<br>138 patients	86 (62,32%)<br>52 (37,68%)	IE, Commando<br>IE, Hemi- Commando
Davierwala PM[17]	Eur J Cardiothorac surg	2020	Retrospective, 1999-2018<br>127 patients	127	IE
Jiang X[23]	J Thorac Dis	2020	Retrospective, 2016-2019<br>14 patients	14	IE

Supplemental Table S4. Authors and description of the techniques

First author	Surgical technique	Mitral valve	Aortic Valve	AMC
David TE[7]	Commando	Mechanical prosthesis (70%)<br>Biological prosthesis (30%)	Mechanical prosthesis (70%)<br>Biological prosthesis (30%)	AMC reconstruction (33, 77%)<br>AMC + mitral anulus reconstruction (10, 23%)
Nilto C. De Oliveira[12]	Commando	Mechanical prosthesis (64%)<br>Biological prosthesis (36%)	Mechanical prosthesis (64%)<br>Biological prosthesis (36%)	Double patch
Su Wan Kim[24]	Commando	Mechanical prosthesis (83,3%)<br>Biological prosthesis (16,7%)	Mechanical prosthesis (83,3%)<br>Biological prosthesis (16,7%)	Aortic annular reconstruction with bovine pericardial strip (2, 6.7%)<br>Mitral annular reconstruction with bovine pericardial strip (2, 6.7%)<br>Aortic and mitral annular reconstruction with bovine pericardial strip (8, 26.7%)
Alberto Forteza[15]	Commando	Mechanical prosthesis (90%)<br>Biological prosthesis (10%)	Mechanical prosthesis (90%)<br>Biological prosthesis (10%)	Double patch
Anton	Hemi	Replacement (7, 20%)	Replacement (100%)	Double patch

Tomšič[22]	Commando/Commando	Repair (28, 80%)		
Elgharably H.[10]	Hemi-Commando	Repair (100%)	Homograft (100%)	Homograft leaflet (30, 81%)<br>Pericardial patch (6, 16%)<br>Dacron patch (1, 3%)
Navia JL[11]	Hemi-Commando	Repair (100%)	Biological prosthesis (3, 5.8%)<br>Allograft (49, 94%)	Pericardial patch (10, 19%)<br>Allograft anterior mitral leaflet extension (42, 81%)
	Commando	Replacement (100%)	Mechanical prosthesis (8, 9.3%)<br>Biological prosthesis (39, 45%)<br>Allograft (39, 45%)	Pericardial patch (64, 74%)<br>Allograft anterior mitral leaflet extension (22, 26%)
Davierwala PM[17]	Commando	Mechanical prosthesis (26, 20.5%)<br>Biological prosthesis (101, 79.5%)	Mechanical prosthesis (26, 20.5%)<br>Biological stented/stentless prosthesis (95, 74.7%)<br>Homograft (6, 4.7%)	Double patch
Jiang X[23]	Hemi Commando/Commando	Repair 6 (42.9%)<br>Mechanical prosthesis 7 (50%)<br>Biological Prosthesis 1 (7.1%)	Mechanical prosthesis 13 (92.9%)<br>Biological prosthesis 1 (7.1%)	Single patch

IE Infective endocarditis; AMC Aortic mitral cortain

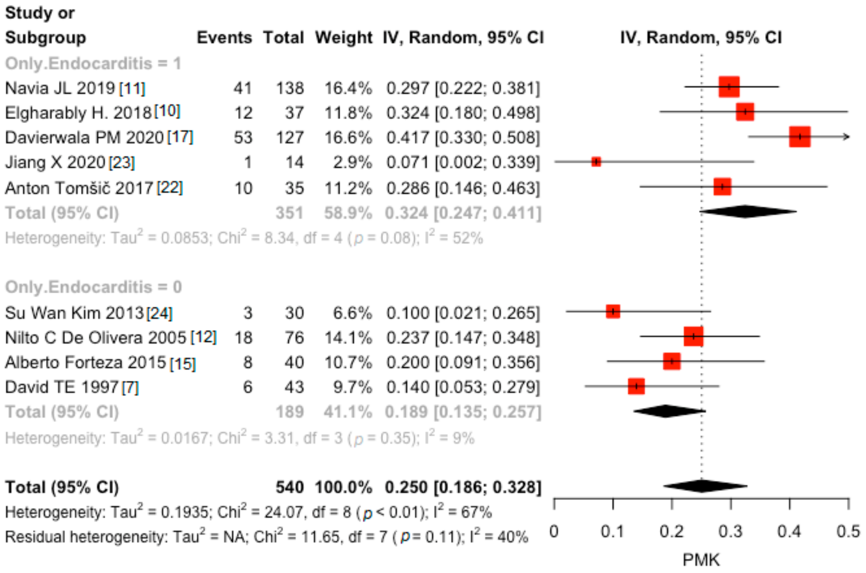

Supplemental Figure S2. Early pacemaker implant by indication to surgery
Pooled proportions of early pacemaker (PM) implant by indications to surgery. Black diamond was the pooled proportion. IV = inverse variance. Proportion is reported on X-axis.
Figure 6

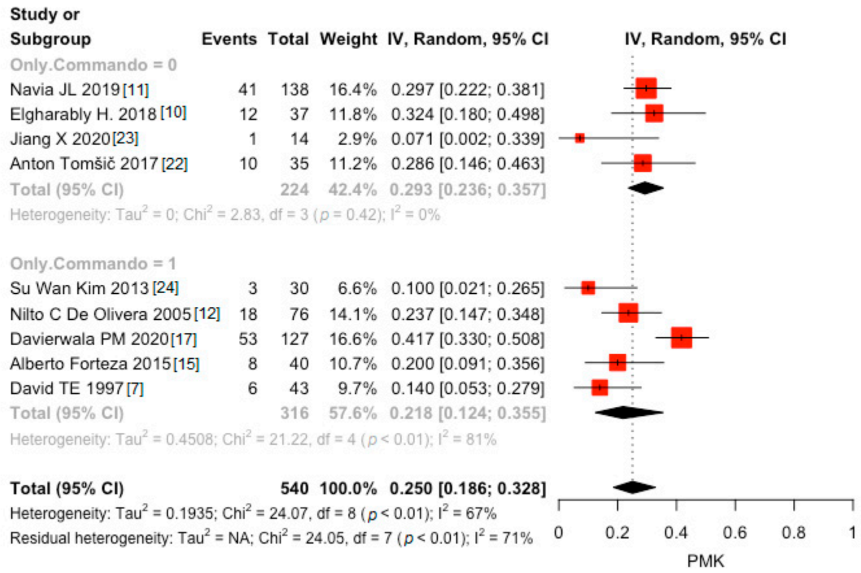

Supplemental Figure S3. Early pacemaker implant by type of prosthesis

Pooled proportions of early PM implant by rate of biological or mechanical prostheses used. Black diamond was the pooled proportion. IV = inverse variance. Proportion is reported on X-axis.

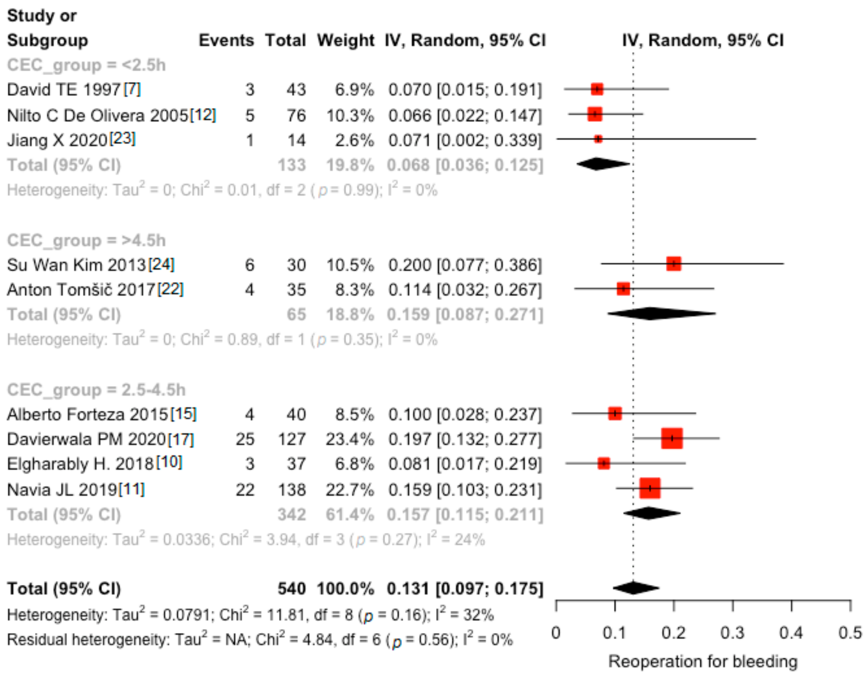

Supplemental Figure S4. Early reoperation for bleeding by Cardiopulmonary bypass time.

Pooled proportions of early reoperation for bleeding by duration of cardiopulmonary bypass (CPB); Black diamond was the pooled proportion. IV = inverse variance. Proportion is reported on X-axis.
